# Eliciting parental preferences and values for the return of additional findings from genomic sequencing

**DOI:** 10.1038/s41525-024-00399-8

**Published:** 2024-02-14

**Authors:** Ilias Goranitis, Yan Meng, Melissa Martyn, Stephanie Best, Sophie Bouffler, Yvonne Bombard, Clara Gaff, Zornitza Stark

**Affiliations:** 1 Health Economics Unit, Centre for Health Policy, Melbourne School of Population and Global Health, University of Melbourne, Melbourne, VIC Australia; 2 Australian Genomics, Melbourne, VIC Australia; 3 Murdoch Children’s Research Institute, Melbourne, VIC Australia; 4 Melbourne Genomics Health Alliance, Melbourne, VIC Australia; 5 Department of Paediatrics, University of Melbourne, Melbourne, VIC Australia; 6 Department of Health Services Research, Peter MacCallum Cancer Centre, Melbourne, VIC Australia; 7 Victorian Comprehensive Cancer Centre Alliance, Melbourne, VIC Australia; 8 Sir Peter MacCallum Cancer Centre, Department of Oncology, University of Melbourne, Melbourne, VIC Australia; 9 Institute of Health Policy, Management and Evaluation, University of Toronto, Toronto, ON Canada; 10 Genomics Health Services Research Program, Li Ka Shing Knowledge Institute of St. Michael’s Hospital, Unity Health Toronto, 30 Bond Street, Toronto, ON Canada

**Keywords:** Health care economics, Genetics research

## Abstract

Health economic evidence is needed to inform the design of high-value and cost-effective processes for returning genomic results from analyses for additional findings (AF). This study reports the results of a discrete-choice experiment designed to elicit preferences for the process of returning AF results from the perspective of parents of children with rare conditions and to estimate the value placed on AF analysis. Overall, 94 parents recruited within the Australian Genomics and Melbourne Genomics programmes participated in the survey, providing preferences in a total of 1128 choice scenarios. Statistically significant preferences were identified for the opportunity to change the choices made about AF; receiving positive AF in person from a genetic counsellor; timely access to a medical specialist and high-quality online resources; receiving automatic updates through a secure online portal if new information becomes available; and lower costs. For AF uptake rates ranging between 50–95%, the mean per person value from AF analysis was estimated at AU$450–$1700 (US$300–$1140). The findings enable the design of a value-maximising process of analysis for AF in rare-disease genomic sequencing.

## Introduction

Genomic sequencing offers an unprecedented opportunity to transform the way healthcare is delivered throughout the human life course^1^. Large-scale genomics research initiatives worldwide have accelerated clinical translation for multiple testing indications, initially in rare disease and cancer^2,3^. Genomic sequencing can also provide information about other conditions that do not relate to the primary indication for testing and may affect health or reproductive choices, called additional (or secondary) findings (AF).

The return of AF remains debated from clinical, ethical, and legal perspectives^4^. The American College of Medical Genetics and Genomics advocates that the return of results from analyses for AF is an almost mandatory part of diagnostic genomic sequencing^5^. The European Society for Human Genetics and the Human Genetics Society of Australasia, however, encourage approaches that minimise the discovery of AF^6^. The key arguments in this debate have mainly focused on the questions *‘Should AF be returned?’* and *‘What AF should be returned?’*. The question of *‘How should AF be returned?’* has attracted limited attention, and little is known about what the key attributes in the process of returning genomic results from AF analyses may be, how people may prioritise these, how much they are valued and the broader welfare implications of a high-valued pathway to returning results from AF analyses.

To maximise the clinical, personal, and economic outcomes of genomic sequencing in the pursuit of high-value, sustainable, and equitable translation, the understanding of the values and priorities of people undergoing diagnostic genomic sequencing is critical. This study aims to elicit preferences for key attributes in the process of returning results from genomic analyses for AF from the perspective of parents with children affected by rare conditions in Australia. Preference-elicitation was undertaken using a discrete-choice experiment (DCE) method^7^. DCEs have been used to elicit preferences and priorities for genomic sequencing in rare conditions and to quantify uptake and willingness-to-pay (WTP)^8–10^, and their use in the area of genomic medicine is increasing^11^. With this study, we provide empirical evidence to support the design of a cost-effective service for returning the results from analyses for AF, and we quantify the uptake and value of returning AF to families with children affected by rare genetic conditions.

## Results

Parents of children with a rare condition recruited within the Australian Genomics (*n* = 492) and Melbourne Genomics Health Alliance (*n* = 190) were invited to participate in the DCE survey. In total, 94 parents completed the survey independently (response rate = 14%). As shown in Supplementary Table 1, the mean age of respondents was 42 years (SD = 7) and the majority of parents were female (90.4%; *n* = 85), married, or in a de facto relationship (83%; *n* = 78), had more than one child (83%; *n* = 78), had private health insurance (63.8%; *n* = 60), lived in metropolitan area (75.5%; *n* = 71) and had received a genomic diagnosis for the child (56.4%; *n* = 53). Parents were interested in an analysis for AF (92.6%; *n* = 87) and especially in AF that included childhood-onset AF for the child (87.3%; *n* = 82). Across all participant responses, AF alternatives were chosen in 70% of the overall choice tasks. Supplementary Fig. 1 shows that most parents were over 90% confident that they would make this choice about receiving AF in real life (65%; *n* = 61). The regression results from the choice analysis are presented in Table 1 and discussed in detail below:

**Table 1:** Marginal utilities and willingness-to-pay (WTP)

Attributes/levels	Mean	Std. error	Importance score, % (ranking)	Marginal WTP, AU$
				Mean (std. deviation)
Receiving additional findings constant	2.54035***	0.52768***		
Opportunity to change choices over time (yes)	0.32148***	0.07484***	6.3% (6)	375 (475)
How positive results are returned^1^				
Directly, telehealth or phone	0.30391	0.20878	16.4% (2)	-
Directly, in person	0.67793***	0.18327		980 (530)
Who returns positive results^2^				
Relevant medical specialist	0.18911	0.16742	12.3% (3)	-
Genetic counsellor	0.43219**	0.19719**		725 (870)
Clinical genetics specialist	0.19636	0.20235		-
How negative results are returned	0.19207	0.12836	3.8% (8)	-
Who returns negative results	−0.00468	0.09514	0.1% (9)	-
Waiting time for seeing a medical specialist (months)	−0.16757***	0.04719***	9.1% (5)	−100 (70)
Immediate access to relevant high-quality online resources	0.32676***	0.05628	6.4% (7)	390 (210)
New relevant information becomes available^3^				
Updates upon individual request	−0.02342	0.09889	9.6% (4)	-
Automatic updates in secure online portal	0.49644***	0.11384		570 (310)
Cost of testing (AU $)	−0.00208***	0.00024***	35.9% (1)	-
Log likelihood function	−724			
McFadden Pseudo R-squared	0.414			
Akaike information criterion	1486			

^***^Statistically significant at 1% level; ^**^Statistically significant at 5% level; ^*^Statistically significant at 10% level.

^1^Marginal utilities and values are relative to the base-level of ‘Electronically through a secure online portal’; ^2^Marginal utilities and values are relative to the base-level of ‘General practitioner’; ^3^Marginal utilities and values are relative to the base-level of ‘No updates will be provided’.

### Preferences for receiving results from analyses for AF

Parents demonstrated a preference for receiving results from analyses for AF, as evidenced by the statistically significant constant term. Preference heterogeneity existed with regards to the level of utility for receiving AF, as evidenced from the statistically significant parameter estimate for the derived standard error of the constant term.

### Preferences for an opportunity to change the choices made about AF over time

Parents on average showed preference for the opportunity to change the choices they made about AF over time, such as the choice to delay the decision to receive AF, receive a different type of AF, or opt out of receiving AF at all. Significant preference variability was identified amongst parents. On average, parents would be willing to pay $375 (US$250) for the opportunity to change the choices made. This attribute was ranked 6th in terms of priority with an importance score of 6.3%.

### Preferences for how the results of additional analysis are returned in the presence of an AF

We found a preference for receiving positive results directly in person compared to an electronic return of results through a secure online portal. No statistically significant difference in the preference for receiving positive results directly through telehealth or phone was identified compared to the electronic return of results. On average, parents would be willing to pay $980 (US$660) to receive the results in person instead of electronically. This attribute was ranked 2nd in terms of priority with an importance score of 16.4%.

### Preferences for who returns the results of additional analysis in the presence of an AF

We found a preference for receiving positive results from a genetic counsellor instead of their general practitioner, and homogeneity in this preference existed. No statistically significant difference in the preference for receiving positive results from a relevant medical specialist or a clinical genetics specialist compared with a general practitioner was identified. On average, parents would be willing to pay $725 (US$485) to receive the results from a genetic counsellor instead of their GP. This attribute was ranked 3rd in terms of priority with an importance score of 12.3%.

### Preferences for how the results of additional analysis are returned in the absence of an AF

No statistically significant preferences were identified with regards to how negative results are returned. This attribute was ranked 8th in terms of priority with an importance score of 3.8%.

### Preferences for who returns the results of additional analysis in the absence of an AF

No statistically significant preferences were identified with regards to who returns negative results. This attribute was ranked 9th in terms of priority with an importance score of 0.1%.

### Preferences for the waiting period until seeing a medical specialist in the presence of an AF

Parents showed negative preference for increased waiting time for seeing a medical specialist. Statistically significant variability existed in the disutility associated with the increased waiting time for seeing the medical specialist. On average, parents would be willing to accept $100 (US$67) for every additional month they need to wait for seeing a medical specialist following the return of a positive AF. This attribute was ranked 5th in terms of priority with an importance score of 9.1%.

### Preferences for accessing relevant high-quality information if there is an additional finding

Positive preference for an immediate access to relevant high-quality online resources was identified, and preferences were homogenous. On average, parents would be willing to pay $390 (US$260) for immediate access to relevant high-quality online resources. This attribute was ranked 7th in terms of priority with an importance score of 6.4%.

### Preferences for what happens if new information relevant to individual results becomes available

Parents demonstrated a preference for receiving automatic updates through a secure online portal compared to not receiving updates. No statistically significant difference existed in the preference between receiving updates upon request and not receiving updates. On average, parents would be willing to pay $570 (US$380) to have automatic updates through a secure online portal if new relevant information becomes available. This attribute was ranked 4th in terms of priority with an importance score of 9.6%.

### Preferences for the cost of the analysis for additional findings to parents

Parents demonstrated negative preference for higher costs. The extent of the disutility associated with the cost attribute varied among parents. This attribute was ranked 1^st^ in terms of priority with an importance score of 35.9%.

Parents with higher household income were found to have significantly higher utility for receiving AF. Living in a metropolitan area, health-related quality of life and having a confirmed genomic diagnosis had no statistically significant effect on the utility of receiving AF. The results in Supplementary Table 2 show that there are two distinct classes of participants with more homogenous preferences. In Class 1, which includes 75% of participants, there is a positive preference towards an analysis for AF (when there are no costs involved and no waiting time to see a medical specialist), with preferences towards the DCE attributes being similar to the ones described above. In Class 2, which includes 25% of participants, there is negative preference towards an analysis for AF, with participants being very price-sensitive and with a strong preference for receiving AF from a genetics specialist.

**Fig. 1:**
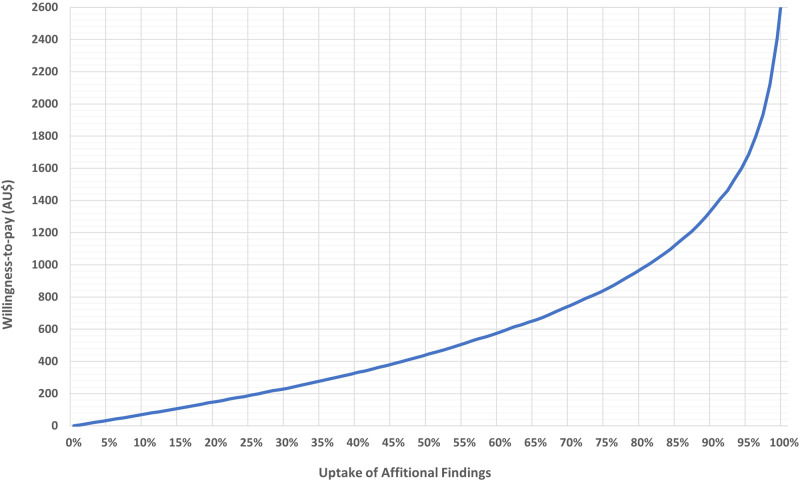
Mean per person value of analysis for additional findings across different uptake thresholds. The figure presents the average willingness-to-pay, in Australian dollars (AU$), for an analysis for additional findings (AF) as a function of the AF uptake percentage among parents.

Based on the preferences of this sample, and with a process of receiving AF in which (a) parents have the option to change the choices made about AF, (b) positive results are returned in person from a genetic counsellor, and (c) there is 5 months of waiting time to see a medical specialist, the mean WTP compared to a context where there is no analysis for additional findings was estimated at $1675 (US$1120). However, not all families would choose to undergo diagnostic genomic sequencing, and amongst those who would, the uptake of analyses for AF may vary. Figure 1 presents the estimated WTP for AF analysis as a function of uptake. For uptake rates ranging between 50–95%, the mean WTP for receiving results from analyses for AF is estimated to range between $450–$1700 (US$300–$1140).

## Discussion

This study elicited preferences for the process of returning results from analyses for AF that are valued by parents with children affected by rare conditions. We identified statistically significant preferences for: an opportunity to change the choices made about AF; receiving positive AF in person from a genetic counsellor; timely access to a medical specialist and high-quality online resources; receiving automatic updates through a secure online portal if new information becomes available; and lower costs. Assuming that the uptake of genomic sequencing for rare conditions affecting children ranges between 60–80%^8^, and the conditional probability of choosing to have AF analysis is 93% (as evidenced in this study), the uptake of AF is expected to range between 56–74%. In this range, the expected WTP for an analysis for AF is estimated to range between $530–$830 (US$355–$556). The marginal values estimated now enable the design and translation of cost-effective processes for analysis and return of AF in Australia through a comparative evaluation of associated costs and the monetary valuations of the benefits that our study estimated. Living in a metropolitan area, parental quality of life and the presence of a confirmed genomic diagnosis for the child did not influence parental preference for AF analysis.

Our study identified a high expected uptake rate of AF (93%). While there may be multiple factors influencing AF uptake rates^12^, emerging evidence suggests that rates may range between 80% and 98%^13–15^. A Canadian study by Regier et al.^16^, elicited societal preferences for the return of AF from genomic sequencing using a discrete-choice experiment method. The study concluded that participants placed, on average an important value (CAN$725; using 2013 as the price-base year) on having a choice about what type of findings they would receive, with a predicted uptake of 76%. In that study, cost was also ranked 1st in terms of priority with a similar importance score (37%) to ours. We identified a preference for receiving automatic updates through a secure online portal if new information becomes available, aligning with the work from Mighton et al.^17^ which identified preference for receiving updates on results through an online database, with the strength of preference varying depending on the certainty of results.

There is also growing interest in the application of online resources to address the unmet information needs of parents with a child affected by a rare condition^18^. Our study contributes to expanding the understanding of the importance of designing value-based online resources in relation to the return of additional findings by not only demonstrating the significance of this attribute but also by quantifying the economic value that it generates, which can support the design of cost-effective strategies for developing online resources. To support the development of sustainable national processes for returning results from analyses for additional findings (AF), it was important to provide empirical evidence on the difference in preferences, priorities, and values between how AF results are returned and by whom when there is an additional finding and when there is not. Our respondents did not find it was important to know how negative AF results are returned and who returns them. This is in contrast to primary findings from genomic tests, where interpretation and perceptions of negative findings in the context of suspected genetic condition is important. Our study identified statistically significant preferences for timely access to medical specialists. This finding supports the importance of designing a whole pathway around the implementation of AF instead of narrowing the focus to the AF analysis and reporting alone. Designing a cost-effective process for returning AF means that intervention-related as well as associated downstream costs and outcomes need to be considered. In this way, it is more similar to considering the design of other screening programs, which take into account workforce and system capacity to manage results quickly and appropriately. Without those broader considerations, a programme that offers AF analysis and result return is likely to be less effective and potentially cause distress.

Our work benefited from a national recruitment of families with children affected by rare conditions through the Australian Genomics and Melbourne Genomics programs. The two-step offer for AF analysis model has been argued to offer the advantage of a temporal separation of the AF offer from the offer of diagnostic testing, which reduces families’ decision burden during a period of high distress, while providing the opportunity to receive more information and reflect on the risks and benefits of the analysis for AF^12,19^. Building on this principle, we provided in-depth information about AF through the adapted version of Genetics Adviser^20^, to further enhance the formation of preferences and support informed choice responses.

Despite these advantages, there are several limitations. The sample size is relatively modest and statistically significant preferences could have been identified in other attributes and attribute levels with a larger sample size. While the survey was designed and validated to minimise participant burden and enable participation regardless of the underlying attitudes towards AF, participation may have been skewed towards those who have a positive preference for AF, or other socio-economic factors, such as educational status, and thus may not be representative on average of the families affected by rare disease. To mitigate those issues, the analysis was weighted to represent the educational status of the Australian population and results were presented in ways that allow broader inferences in terms of AF uptake.

The time between genomic testing and the return of primary genomic results is an attribute with demonstrated value to patients and the public^9,10^. In the context of AF analysis, however, our attribute development process supported that the timing to results would not be an attribute of priority compared to the included attributes and that participants interested in AF analysis would take up the analysis regardless of minor variations in months for the return of results. Given the large number of attributes included in this study, we therefore assumed that known monthly variations to the time between AF analysis and results would not lead, on average, to significant reduction in the utility for receiving AF. Nevertheless, we acknowledge that this attribute may still be valued by some individuals. In the process of establishing standardised and nationally scalable processes for returning AF, clinician preferences may have an important role to play^21^, and further research in this space is needed to understand alignment between patient and clinician preferences. Finally, one of the objectives at the conceptualisation of this work was to explore differences in the relative attribute importance and overall value of receiving AF between the different types of AF (i.e., childhood-onset AF for the child, adult-onset AF for the parent, and genetic carrier screening for the couple). However, the modest sample size (*n* = 94) and the small number of respondents (*n* = 5) being interested in an adult-onset AF or couples carrier screening without childhood-onset AF meant that this was not possible. Further research exploring how preferences, values and priorities may differ depending on the type of AF is needed.

In conclusion, our study elicited preferences for the process of returning AF from the perspective of parents with children affected by rare conditions and enabled an estimation of the value that parents attach to the opportunity of having AF analysis. The evidence generated can inform the development of service delivery models for the implementation of AF analyses into clinical care and enable cost-benefit analyses to optimise the health economic outcomes derived from AF analyses. Our findings provide useful economic insights into the discourse around the return of AF, and it is pertinent that health economic evidence related to the analysis and return of AF continues to develop to support the cost-effective integration of genomic medicine in healthcare systems.

## Methods

### Ethics statement

Informed consent for participating in the study and publishing the study findings was obtained electronically from all participants prior to entering the survey by selecting the option *“I consent to participate in this survey as outlined in the Plain Language Statement”*. Ethics approval was granted from the Medicine and Dentistry Human Ethics Sub-Committee of the University of Melbourne (Reference Number: 2022-23928-34604-4). The study complied with all relevant ethical regulations including the Declaration of Helsinki.

### Study design and participants

Parents of children with a suspected rare condition were recruited into disease-specific flagship studies (e.g., brain malformations, cardiomyopathies, renal conditions, immunodeficiencies) through Australian Genomics^2,3^ and Melbourne Genomics Health Alliance^22^ between 2016–2021. For this survey, parents of children recruited into rare-disease flagships who had consented to secondary research as part of their original research consent were invited to participate, in line with the two-step offer and return of additional genomic findings model, which separates AF analysis from the initial episode of diagnostic testing^12,19^. Parents were not offered to receive AF when they had the testing done for their child. Invitations were coordinated by the data management teams at Australian Genomics and Melbourne Genomics. Email invitations were sent to eligible parents, followed by a single reminder 6–10 weeks after the initial invitation. All survey responses were anonymous. The DCE survey was designed based on good research practice recommendations^23^, and can be accessed through the online supplementary material. The first section of the survey collected sociodemographic information and information on whether families had received a genomic diagnosis. The second section invited participants to view an online video about AF analysis. Following the video, the survey provided information about the three types of AF: (a) Childhood-onset additional findings for the child; (b) Adult-onset additional findings for the parent; and (c) Genetic carrier (reproductive) screening for the couple. The survey then listed risks and benefits from the return of AF with respect to treatment, future health conditions, personal and family impacts. The background information on AF represented a modified version of Genetics Adviser^20^, developed for the Australian Genomics Acute Care Genomics Programme Additional Findings substudy^12^.

The third section of the survey reminded participants about the three types of AF, what they may learn, why it may be helpful and whether they could take action based on the AF. Participants were then asked to make a hypothetical choice about the type(s) of AF they were interested in and indicate how certain they were about making this choice in real life. For participants who were not interested in AF the survey ended in section 3 for pragmatic reasons related to reducing any likely burden to these families. Participants interested in AF proceeded in the fourth section of the survey, which provided information about key attributes in the process of returning AF. The attributes were developed based on a review of the literature^4^, review of survey results (*n* = 93) and interview transcripts (*n* = 14) with healthcare professionals as part of a study exploring the acceptability and feasibility of offering AF, review of data from a process evaluation of an AF service conducted by the Melbourne Genomics Health Alliance (specifically interviews with participating genetic counsellors (*n* = 6) and a focus group with medical scientists (*n* = 10) performing the tertiary AF analysis), and expertise drawn from the Australian and Melbourne Genomics Health Alliance teams^19^, and are listed in Table 2.

**Table 2:** Attributes and attribute levels

Attributes	Levels
1. Opportunity to change the choices you made about additional findings over time^**1**^	No
	Yes
2. How the results of additional analysis are returned to you when there is an additional finding	Electronically through a secure online portal
	Directly, telehealth or phone
	Directly, in person
3. Who returns the results of additional analysis to you when there is an additional finding (relevant only when results are returned directly)	Noone
	Your general practitioner
	A relevant medical specialist
	A genetic counsellor
	A clinical genetics specialist
4. How the results of additional analysis are returned to you when there is NOT an additional finding	Electronically through a secure online portal
	Directly, telehealth or phone
	Directly, in person
5. Who returns the results of additional analysis to you when there is NOT an additional finding (relevant only when results are returned directly)	Noone
	Your general practitioner
	A relevant medical specialist
	A genetic counsellor
	A clinical genetics specialist
6. How long is the waiting period for seeing a medical specialist if there is an additional finding	2 weeks
	2 months
	6 months
7. Options for accessing additional information if there is an additional finding	Additional online information is not available
	You will be given immediate access to relevant high-quality online resources
8. What happens if new information relevant to your results becomes available	No updates will be provided automatically
	Updates will be provided upon request
	Updates will be provided automatically in a secure online portal
9. Cost of testing to you (AU$)	250
	500
	1000
	2000

^1^such as the choice to delay the decision to receive additional findings (AF), receive a different type of AF, or opt-out of receiving AF at all.

The fifth section asked participants to make choices based on how important these characteristics were to them. Choice tasks were developed based on a D-efficient experimental design including 24 choice tasks with no overlapping attributes, split into 2 blocks (i.e., 12 choice tasks per participant), using Ngene (ChoiceMetrics Pty Ltd, Sydney, NSW, Australia). Blocking was performed to reduce task effort for the respondent and was implemented using the minimum correlation principle. Participants were randomised to one of the two blocks. Four rounds of piloting with Australian Genomics study participants (*n* = 17) were performed before the full launching of the survey to explore whether participants had difficulty in understanding attribute descriptions or completing the choice tasks, and to ascertain expected response rate and possibility for redesign to account for likely interactions, e.g., between who returns the results and how. An example of a choice task, which was presented in the original survey as a “warm-up” task before participants entered the choice experiment, is available in the online supplementary material.

### Choice data analysis

The choice dataset included choice data from respondents interested in AF (completed sections 1–5) and imputed opt-out choice data from those who were not interested in AF (completed sections 1–3). A mixed multinomial logit model was estimated using NLOGIT 6 (Econometric Software, Inc., Waverton, NSW, Australia) to accommodate unobserved preference heterogeneity around the return of AF^7^. Preference heterogeneity is incorporated using random parameters with a specified probability distribution. The model was incrementally built and tested for preference heterogeneity, predictive ability, and goodness of fit. The final model included normal distributions for Attributes 1, 3 and the alternative specific constant for the choice of receiving AF, and a constrained triangular distribution for the cost and time attributes (Attributes 6 and 9). Random parameters were estimated using 500 standard Halton draws. Effects coding was used except for the cost and time attributes as well as for attributes related to the return of negative AF, where continuous coding was used. The primary analysis relied on a main effects model specification of the DCE attributes. Given the highly educated sample of participants and the known positive effect of education on genomic sequencing uptake and valuation^8^, the data were exogenously weighted by education according to the 2021 Australian census^24^. An additional analysis incorporated income level, living in a metropolitan area, health-related quality of life, and the presence of a confirmed genomic diagnosis via main effects in the utility function. Marginal WTP values for the DCE attributes were estimated using the unconditional population moments estimates^7^. Simulated marginal WTP values were reviewed for face-validity independently by the study team. The 0.10 percentile appeared to reflect the cut-off point and was used to exclude extreme values from the mean and standard deviation estimation. The relative importance of each attribute was estimated using importance scores, calculated by comparing the range in estimated utility between best and worst attribute levels divided by the sum of the utility ranges across attributes^9,10^. The incremental WTP for receiving AF was then calculated using the compensating variation formula^25^. The equivalent formulation based on probabilities^26^, was used to graph WTP as a function of AF uptake given that the study elicited preferences from participants already recruited within genomic studies and DCE survey participation may be indicative of prior positive attitudes to the return of AF. WTP values are reported in both Australian and US dollars (using 1 July 2023 Reserve Bank of Australia exchange rate of 0.67).

### Reporting summary

Further information on research design is available in the Nature Research Reporting Summary linked to this article.

### Supplementary information

Supplemental Material

REPORTING SUMMARY

## Data Availability

Choice data are available from the corresponding author upon reasonable request.
