# Supplementary material for: Eliciting parental preferences and values for the return of additional findings from genomic sequencing
Source: NPJ Genom Med. 2024 Feb 14;9:10. doi: 10.1038/s41525-024-00399-8 (PMC10867021; doi:10.1038/s41525-024-00399-8)
Supplement: Supplementary file 1 — Supplemental Material [file 41525_2024_399_MOESM1_ESM.pdf]

Supplementary Table 1 Personal characteristics

Sociodemographic variables	n (%)
**Age**	42 years (SD = 7)
**Gender (Female)**	85 (90.4)
**Marital status**	
Married or in a de facto relationship	78 (83)
**Educational status**	
Higher level education (University degree or higher)	38 (40.4)
**Annual household income**	
Lower than $40,000 per year	5 (5.3)
$40,000 - $60,000 per year	11 (11.7)
$60,000 - $80,000 per year	14 (14.9)
$80,000 - $100,000 per year	10 (10.6)
$100,000 - $120,000 per year	11 (11.7)
$120,000 - $140,000 per year	9 (9.6)
$140,000 - $160,000 per year	8 (8.5)
Over $160,000 per year	26 (27.7)
**Children (>1 = Yes)**	78 (83)
**Private health insurance (Yes)**	60 (63.8)
**Live in Metropolitan area (Yes)**	71 (75.5)
**Health score (0-1)**	0.76 (SD = 0.18)
**Have received a genomic diagnosis for the child**	53 (56.4)
**Type of Additional Findings (AF) selected**	
No AF	7 (7.4)
Adult-onset AF for the parent	2 (2.1)
Genetic carrier screening for the couple	2 (2.1)
Adult-onset AF for the parent and Genetic carrier screening for the couple	1 (1.1)
Childhood-onset AF for the child	15 (16)
Childhood-onset AF for the child and Adult-onset AF for the parent	30 (31.9)
Childhood-onset AF for the child and Genetic carrier screening for the couple	3 (3.2)
All AF	34 (36.2)

Supplementary Table 2 Marginal utilities based on the latent class choice model

**Attributes/Levels**	**Class 1**	**Class 2**
Receiving additional findings constant	2.77513***	-1.84954**
Opportunity to change choices over time (Yes)	0.24200***	1.11634***
How positive results are returned		
Directly, telehealth or phone	0.37076**	-0.68486
Directly, in person	0.61867***	0.74089
Who returns positive results		
Relevant medical specialist	0.11924	-0.39697
Genetic counsellor	0.30626*	-0.07520
Clinical genetics specialist	-0.04044	1.55247**
How negative results are returned	0.08331	0.48298
Who returns negative results	-0.07899	-0.19965
Waiting time for seeing a medical specialist (months)	-0.13758***	-0.07120
Immediate access to relevant high quality online resources	0.24948***	0.52835**
New relevant information becomes available		
Updates upon individual request	-0.10210	0.36295
Automatic updates in secure online portal	0.39121***	-0.11698
Cost of testing (AU $)	-0.00094***	-0.00315***
Class probabilities	0.75	0.25
Log likelihood function	-783	
McFadden Pseudo R-squared	0.37	
Akaike information criterion	1623	

*** Statistically significant at 1% level; ** Statistically significant at 5% level; * Statistically significant at 10% level

Supplementary Figure 1 Distribution of parents depending on their certainty in taking up analysis for additional findings (AF) in real life

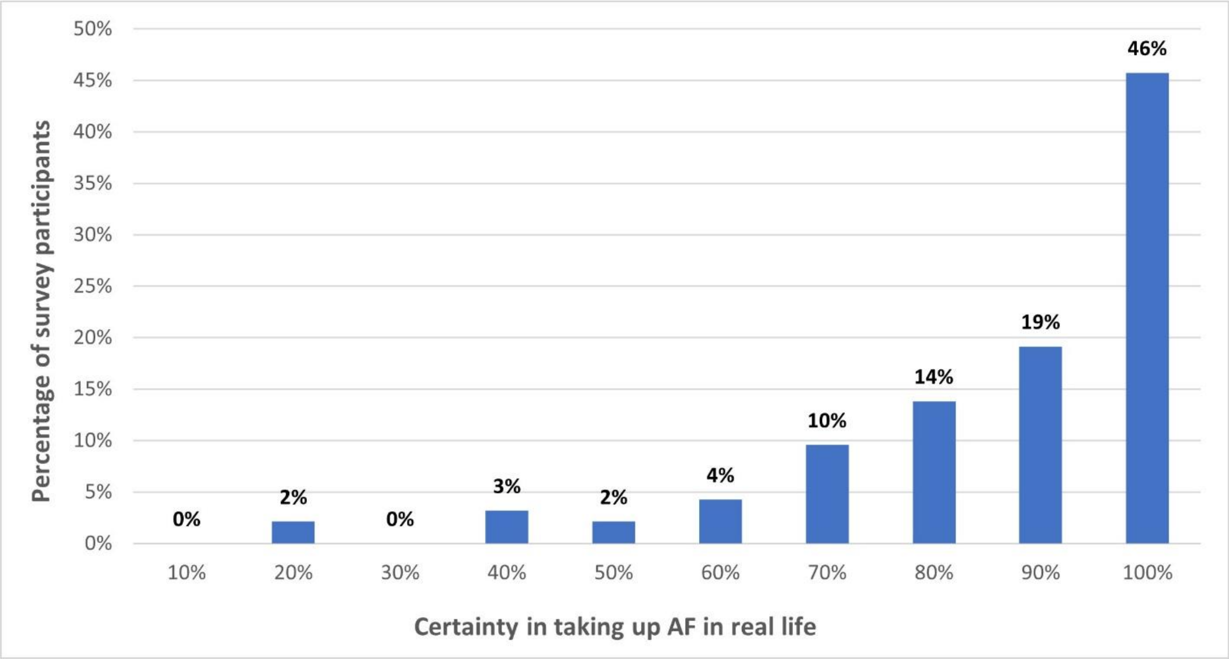

Preferences for the return of additional genomic findings

As part of the Australian/Melbourne Genomics Program you had a genomic sequencing test for your child's medical condition. The test aimed to identify a genetic cause for the condition to inform treatment or management.

Following genomic testing the data are held securely by the testing laboratory. It is possible to look at these data again for information about other conditions, not related to the reason for the original test, that may affect people's health or future children. This is called *'analysis for additional findings'*.

With this survey, we want to understand how to best return results of analysis for additional findings to individuals or families.

Please note that we are NOT currently offering you analysis for additional findings. We only want your opinion about what types of additional findings might be of interest to you and how you imagine you would like to receive the results in a hypothetical situation.

The survey will take about 30 minutes to complete.

Your participation is voluntary. Your responses will be confidential and will not be shared with anyone outside the research team. This research has ethical approval from the Medicine and Dentistry Human Ethics Committee of the University of Melbourne. You can withdraw from this research at any time and without any consequences.

In the following page, we provide a description of the project and the nature of your participation.

To enter the survey, you will need to provide your consent. Your consent will allow us to use the information you provide for our research.

If you have any questions, please contact A/Prof Ilias Goranitis, University of Melbourne, at: ilias.goranitis@unimelb.edu.au.

We appreciate your time and support.

Plain Language Statement

Centre for Health Policy/ Melbourne School of Population and Global Health

Project: Preferences for the return of additional genomic findings

A/Prof Ilias Goranitis; Tel: +61 3 834 49959; Email: ilias.goranitis@unimelb.edu.au

Introduction

We are contacting you because you told us you might be willing to participate in further research conducted by the Australian/Melbourne Genomics Health Alliance. The following few pages will provide you with further information about this survey, so that you can decide if you would like to take part in this research.

Please take the time to read this information carefully. You may ask questions about anything you don't understand or want to know more about. Your participation is voluntary. If you don't wish to take part, you don't have to. If you begin participating, you can also stop at any time. This research is funded by a Medical Research Future Fund (MRFF) Genomics Health Futures Mission grant titled "National rapid genomic diagnosis program for critically ill children".

What is this research about?

Genomic sequencing writes out your whole genetic code (the genome). This can be read (analysed) for many purposes, typically to identify the cause of a medical condition and inform treatment. The data can also be reanalysed to uncover other types of information, such as health information that is unrelated to the reason for the original test ('additional findings'). This information may be useful to individuals; for example, to prevent or treat another condition. However, reporting these 'additional findings' is not routine, and professionals disagree about if and how it should be done. With this research, we want to understand your views about analysis for additional findings and the key issues that are important to you. This will help inform how 'additional findings' are made available in the future.

What will I be asked to do?

If you agree to participate, you will be asked to complete one survey. The survey will take up to 30 minutes. Initially, the survey asks questions about you and your experience of genomic testing. You will then be provided with information about the analysis for additional findings, and we will ask you if you would be potentially interested in such additional analysis. **Please note that this question is hypothetical**. Nevertheless, we would really appreciate an honest answer. Those who would potentially be interested in an analysis for additional findings, will be asked to make some choices based on how they would prefer to receive the additional information.

What are the possible benefits?

There will be no direct benefits from your participation. The findings from this work will enable us to design a process for returning additional information that reflects people's preferences and values.

What are the possible risks?

We do not anticipate any risks in participating in this study. It may be that taking part may cause you to think about your or your family's wellbeing or reflect on the need for an analysis for additional findings. If you want more information about an analysis for additional findings, please contact your genetic counsellor. If you have any concerns about your health, please seek advice from your GP.

Do I have to take part?

No. Participation is completely voluntary, and you can withdraw at any time before completing the survey and without any consequences. Please note that once you complete the survey, it is not possible to withdraw from the study because data are anonymised and not identifiable.

Will I hear about the results of this project?

A summary of the survey findings will be placed on the Australian Genomics and the University of Melbourne websites. We will also be sharing the results of the survey more widely via academic journals and conferences, and through the development of a summary to distribute to patient advocacy groups, policy makers, and health providers.

What will happen to information about me?

All the information gathered from the surveys will be treated confidentially. Survey data will be held on University of Melbourne computers and retained for 5 years following the publication of our findings.

Who is funding this project?

This project is being funded by the Medical Research Future Fund's (MRFF) Genomics Health Futures Mission (GHFM76747). The funding agreement presents researchers' independence in designing the study, conducting the research, interpreting the data, writing, and publishing the findings.

Where can I get further information?

If you would like more information about the project, please contact the researchers;

A/Prof Ilias Goranitis +61 3 834 49959 Email: ilias.goranitis@unimelb.edu.au

Prof Zornitza Stark Tel: +61 3 834 16368 Email: zornitza.stark@vcgs.org.au

You can keep a copy of this form for your records.

Who can I contact if I have any concerns about the project?

This research project has been approved by the Human Research Ethics Committee of The University of Melbourne. If you have any concerns or complaints about the conduct of this research project, which you do not wish to discuss with the research team, you should contact the Manager, Human Research Ethics, Research Ethics and Integrity, University of Melbourne, VIC 3010. Tel: +61 3 8344 2073 or Email: HumanEthics-complaints@unimelb.edu.au. All complaints will be treated confidentially. In any correspondence, please provide the name of the research team or the name or ethics ID number of the research project.

Consent

Do you consent to participate in this survey as outlined in the Plain Language Statement?

By clicking yes, you provide your consent to use survey responses for the purposes of our study.

Please remember that you can withdraw from the study at any point before the survey is completed. As soon as the survey is completed, we are not able to identify your responses because the data are anonymised and not identifiable.

☐ Yes, I consent to participate in this survey as outlined in the Plain Language Statement.

☐ No, I do not wish to consent to participate in this survey.

Section 1

1. How old are you? __________________ years

2. What is your gender?

   ☐ Female ☐ Male ☐ Prefer to self-describe

3. What is your current marital status?

   ☐ Never married

   ☐ De facto (living with a partner)

   ☐ Married

   ☐ Widowed

   ☐ Divorced / separated

   ☐ Other (please specify): __________________

4. What is your highest level of education?

   ☐ Year 11 or below

   ☐ Year 12 or equivalent

   ☐ Certificate

   ☐ Diploma/advanced diploma

   ☐ Bachelor's degree

   ☐ Graduate diploma/certificate

   ☐ Post-graduate degree

   ☐ Other (please specify): __________________

5. What is your household's annual gross (before tax) income?

   ☐ Lower than $40,000 per year

   ☐ $40,000 - $60,000 per year

   ☐ $60,000 - $80,000 per year

   ☐ $80,000 - $100,000 per year

   ☐ $100,000 - $120,000 per year

   ☐ $120,000 - $140,000 per year

   ☐ $140,000 - $160,000 per year

   ☐ Over $160,000 per year

6. Do you have private health insurance?
   ☐ Yes
   ☐ No

7. What is your place of residence?
   ☐ Metropolitan
   ☐ Non-metropolitan

8. How many children do you have? ________

9. How many of your children are 15 years or younger? _______

10. How is your own health today?
    Give a number between 0 (worst health you can imagine) to 100 (best health you can imagine): ___________

11. In which of the following programs did you participate?
    ☐ Acute care genomics
    ☐ Mitochondrial disorders
    ☐ Developmental epileptic encephalopathy
    ☐ Leukodystrophy
    ☐ Malformations of cortical development
    ☐ Cardiovascular genetic disorders
    ☐ Genetic immunology
    ☐ Interstitial and diffuse lung disease in children (chILDRANZ)
    ☐ Neuromuscular disorders
    ☐ Renal genetic disorders
    ☐ Intellectual disability
    ☐ HIDDEN renal genetic disorders
    ☐ Complex care
    ☐ Deafness
    ☐ Dialated cardiomyopathy
    ☐ Immunology
    ☐ Complex neurological and neurodegenerative diseases
    ☐ Bone marrow failure
    ☐ Other (please specify): __________________

12. Did you receive a genetic diagnosis?

☐ No

☐ Yes

☐ I am not sure

Section 2

What is an analysis for additional findings:

https://youtu.be/SXi6-tlFVfw

There are 3 types of additional findings. Please read the information below carefully.

Childhood-onset additional findings for my child

Childhood-onset additional findings are results that tell you about health conditions that might occur in your child during childhood. These may or may not have a known treatment or intervention to improve your child's health

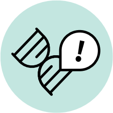

Examples

Muscular dystrophy, some types of blindness or deafness.

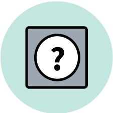

Analysis for childhood-onset additional findings can reveal your child's chance of developing another genetic condition.

Genetic conditions may be severe or mild. Some individuals may not have any symptoms at the time they have analysis for additional findings. They may or may not develop the health conditions.

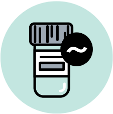

Most of these genetic conditions are not preventable.

There may or may not be treatments to help with the symptoms.

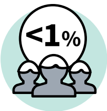

Only a small number of children will have a childhood-onset additional finding.

Fewer than 1 child out of 100 (less than 1%) will find they have one of these DNA changes.

Adult-onset additional findings for myself

Adult-onset additional findings are results that tell you about health conditions that may be detected, prevented, or managed by screening tests, medication, or surgeries. Adult-onset additional findings are only offered to parents and not to your child. Each parent can decide whether or not they want this information for themselves.

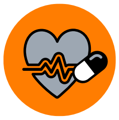

Genetic testing may reveal risks for health conditions that can be managed or prevented.

Some conditions have available medical treatments such as screening, medications, or surgeries to try to manage or prevent the condition.

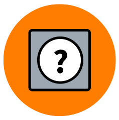

Examples

Hereditary breast and ovarian cancer, hereditary bowel cancer, genetic heart conditions.

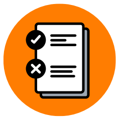

Not all medically actionable conditions are included.

Genetic testing is not a perfect science - it cannot test for all medically actionable conditions.

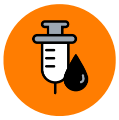

You may need more tests and procedures.

If you are found to have an adult-onset additional finding, you may need to take more tests or have follow up procedures that could be invasive. You may or may not develop the health condition in the future.

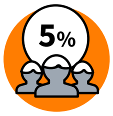

It is uncommon to have an adult-onset additional finding.

5 people per 100 (5%) will find that they have one of these DNA changes.

Genetic carrier screening (reproductive) for us as a couple

Carrier results are DNA changes that don't usually affect your own health but may affect your future children, their children or other family members. Genetic carrier screening is only offered to parents and not to children. Both parents must agree whether or not they want this information as a couple.

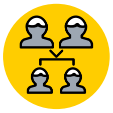

Examples

Carrier for cystic fibrosis, sickle cell disease, some forms of developmental delay.

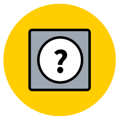

Carrier results are DNA changes that do not affect you, but may affect your future children.

Being a carrier means you most likely won't have any symptoms because you only carry one copy of a DNA change for a health condition. Very rarely, carriers have mild features of some conditions. But, if the analysis shows both parents are a carrier for the same genetic condition, then your children could develop the condition.

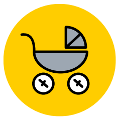

Carrier status can impact decisions to have a child.

The children of two carriers of the same health condition are at an increased risk of developing that condition.

Knowing in advance that both parents are carriers of the same genetic condition may be helpful. Some parents may wish to undergo testing during pregnancy or pre-implantation genetic testing, using IVF, to avoid having a child with the genetic condition. Others may prefer not to use these options but wish to know this information in advance to help them plan.

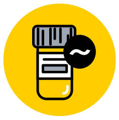

Some of these conditions are treatable, others are not.

Most of these conditions cannot be cured, but some conditions have medications or diet restrictions that can help prevent the condition or reduce its symptoms.

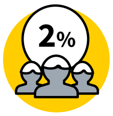

A couple's result will be provided

2 out of 100 couples (2%) will have an increased chance of having a child with another genetic condition

Important things to think about

There are many important things to consider when it comes to deciding about whether to receive 'additional findings' — here are some topics to think about.

Benefit	Risk
**Treatment**	
Results may help your doctor decide on the best management for a health condition you may be at risk of.	There may be no way to treat or prevent some health conditions.
**Future health conditions**	
You may learn about the chance of future health conditions that may affect you and your family members.	Chances are only estimates. It is uncertain if or when you will develop symptoms, or which symptoms you may develop.
**The additional findings analysis**	
Analysis for additional findings looks for many health conditions.	Analysis for additional findings is not a perfect science — it does not test for all health conditions.
**Impact on you**	
Knowing about the chance of health conditions early might help with life planning or symptom management.	In some cases, the results may make you worry and watch for symptoms of a condition.
**Impact on family**	
Knowing about your chance of a condition may also help your family members, as they may have the same additional finding as you.	Some family members may not want to know this information.

Content taken from a modified version of Genetics Advisor developed for the Australian Genomics Acute Care Genomics Program in collaboration with Dr. Yvonne Bombard and team.

Section 3

Genomic testing can be performed either in the affected child alone, or together with the parents. This is known as trio testing. For the purpose of this survey, please assume that testing has been performed on the child together with both parents, so data are available on all three. Now, suppose that you are offered an analysis for additional findings and a genetic counsellor will be there with you to assist you with any additional questions.

Here is a recap

TYPE OF RESULT	What might I learn?	Why it might be helpful	Can I act on it?*
Childhood-onset additional findings	Conditions that have a high chance of causing mild to severe symptoms in my child, many of which may have no known treatment or prevention, e.g., muscular dystrophy, blindness	For life-planning, symptom management	No, but there may be treatments to help reduce or manage symptoms
Adult-onset additional findings	Conditions that have a high chance of medically actionable findings in myself, e.g., breast cancer	To reduce risk/delay onset of health condition	Yes, with screening, medications, surgery, etc.
Genetic carrier screening for couples	Carrier for conditions that may not affect each parent but could affect future children or other family members, e.g., cystic fibrosis	For family planning	Yes, with prenatal testing or other reproductive options

* These actions are generalisations, but there are exceptions, and medical options will change over time.

From the list of possible analyses for additional findings shown below, please indicate which additional findings you want. You can 'tick' all that apply or select 'None at all'.

- ☐ Childhood-onset additional findings for my child.
- ☐ Adult-onset additional findings for myself.
- ☐ Genetic carrier screening (reproductive) for us as a couple.
- ☐ None at all.

How certain are you that you would make this choice in real life?

Please choose a number between 1 (Not confident at all) to 10 (Really confident): ___________________

Section 4

Knowing that you are interested in an analysis for additional findings, this section provides information about key characteristics in the process of returning these results to you. Please read carefully as the next section will ask you to make choices based on how important these characteristics are to you.

1. Having an opportunity to change the choices you made about additional findings

This characteristic tells you whether you have an opportunity to change any choice that you have made about additional findings over time. This may relate to one or more of the following choices:

- Choice to delay your decision to receive the results of the additional analysis to a later time,
- Choice to receive a different type of analysis for additional findings,
- Choice to opt-out of receiving additional findings at all.

2. How the results of additional analysis are returned to you

This characteristic tells you whether you will receive your results from the additional analysis directly from a health professional or electronically through a secure online portal.

- Receiving results directly from a health professional allows you to ask questions and get a good understanding of genomic information. You can receive your results directly either in person or through phone or telehealth, depending on your preferences.
- Receiving results directly through a secure online portal allows you to see the results as soon as they are available and gives you control over your own results, including who you discuss them with and when.

3. Who returns the results of additional analysis to you

If you would like to receive your results from the additional analysis directly from a health professional, this characteristic tells you who will be responsible for returning the results to you.

- Some people prefer to receive genomic results from clinical genetics specialists because of their expertise in genomic sequencing and their ability to interpret the results in a clear way.
- Other people may prefer to receive the results from a genetic counsellor because of their experience in supporting people through the process of receiving genomic results or additional findings.
- Other people may prefer to receive the results from a relevant medical specialist, as they are more interested in the medical implications of their results.
- Others may prefer to receive the results from their general practitioner because they feel more comfortable with discussing personal information.

4. How long is the waiting period for seeing a medical specialist if there is an additional finding

Following an additionalfinding, you may be referred, for example, to a cardiologist or a cancer specialist. However, there is usually a waiting period to see medical specialists. Receiving additional findings may have an emotional impact, and people may not want to delay discussing their findings and the appropriate treatment or preventive options for them. This characteristic tells you how long you may need to wait until seeing a medical specialist. This may range from a few days to a few months depending on the capacity of the system.

5. Options for accessing further online information if there is an additional finding

An additional finding may cause uncertainty to families, and people may want to ask questions and learn more about their findings and the potential implications for their health. Having access to good quality online resources may be important, particularly during the waiting period for seeing a medical specialist. This characteristic tells you whether you will be given immediate access to relevant high quality online resources.

6. What happens if new relevant information about additional findings becomes available?

Over time, new information may become available related to your results from the analysis for additional findings. For example, more additional findings relevant to your or your family's health may become available as a result of improvements in knowledge or changes in clinical practice. This characteristic tells you what happens if new relevant information about additional findings becomes available. At the moment, no updates are provided automatically. However, there is an opportunity to provide an update upon individual request or to provide ongoing updates through a secure online portal.

7. Cost of testing to you

Because the analysis for additional findings is new, there is no Government funding. This characteristic tells you how much you would need to pay out-of-pocket (personally) for the test. The cost could range from $250 to $2000. The analysis for additional findings will be performed once and the payment will be a one-off payment.

Please note:

The survey does not require you to actually pay for the test.

However, to help us accurately value the analysis for additional findings, please pay attention to the actual costs of the test presented to you and carefully consider whether this is an amount you would be willing to pay if you were offered an additional analysis of genomic data.

Remember that this amount would no longer be available for you to spend on other things, such as grocery or utility bills.

Section 5

CHOICE TASKS Example

Which process of returning additional findings would you prefer?

You can choose either 'Process 1', 'Process 2', or 'None of them'

Process characteristics	Process 1		Process 2	
Opportunity to change the choices you made about additional findings over time	Yes		No	
How the results of additional analysis are returned to you	If **there is** additional finding	If **there is** NO additional finding	If **there is** additional finding	If **there is** NO additional finding
	Directly, in person	Directly, telehealth or phone	Directly, telehealth or phone	Directly, telehealth or phone
Who returns the results of additional analysis to you	If **there is** additional finding	If **there is** NO additional finding	If **there is** additional finding	If **there is** NO additional finding
	A genetics specialist	A genetics specialist	A genetics specialist	A genetics specialist
How long is the waiting period for seeing a medical specialist if there is an additional finding	2 months		2 weeks	
Options for accessing additional online information if there is an additional finding	Additional online information is not available		Additional online information is not available	
What happens if new relevant information about additional findings becomes available?	Updates will be provided upon request		Updates will be provided upon request	
Cost of testing to you	$1000		$250	

I would choose: ☐ Process 1 ☐ Process 2 ☐ None of them

In the above example, Process 1 was $750 more expensive than Process 2 and had 1.5 months longer waiting period for seeing a medical specialist if there is an additional finding. However, in Process 1 the results of the additional analysis are returned to you in person instead via telehealth or phone and you have the opportunity to change the choices you made about additional findings over time. Process 1 and 2 are similar in terms of the remaining characteristics. If you are interested in an analysis for additional findings and you believe that the additional cost and waiting time in Process 1 are justified for the opportunity to receive your results in person and the flexibility of changing your choices over time, then you should select 'Process 1'. If you believe that the lower cost of Process 2 and the more timely access to the medical specialist are more important to you than the in-person return of additional findings, then you should select 'Process 2'. If none of these processes reflect your preferences, please select 'None of them'.
